# Geographic patterns in morphometric and genetic variation for coyote populations with emphasis on southeastern coyotes

**DOI:** 10.1002/ece3.4966

**Published:** 2019-02-21

**Authors:** Joseph W. Hinton, Elizabeth Heppenheimer, Kyla M. West, Danny Caudill, Melissa L. Karlin, John C. Kilgo, John Joseph Mayer, Karl V. Miller, Margaret Walch, Bridgett vonHoldt, Michael J. Chamberlain

**Affiliations:** ^1^ Warnell School of Forestry and Natural Resources University of Georgia Athens Georgia; ^2^ Department of Ecology and Evolutionary Biology Princeton University Princeton New Jersey; ^3^ Tacoma Washington; ^4^ Florida Fish and Wildlife Conservation Commission Gainesville Florida; ^5^ Department of Physics and Environmental Sciences St. Mary's University San Antonio Texas; ^6^ United States Department of Agriculture Forest Service Southern Research Station New Ellenton South Carolina; ^7^ United States Department of Energy, Environmental Sciences, and Biotechnology Savannah River National Laboratory Aiken South Carolina; ^8^ Western Biological LLC Elko Nevada; ^9^ Present address: Alaska Department of Fish and Game Fairbanks Alaska

**Keywords:** *Canis latrans*, colonization, coyote, dispersal, genetics, geographic variation, morphometrics, range expansion

## Abstract

Prior to 1900, coyotes (*Canis latrans*) were restricted to the western and central regions of North America, but by the early 2000s, coyotes became ubiquitous throughout the eastern United States. Information regarding morphological and genetic structure of coyote populations in the southeastern United States is limited, and where data exist, they are rarely compared to those from other regions of North America. We assessed geographic patterns in morphology and genetics of coyotes with special consideration of coyotes in the southeastern United States. Mean body mass of coyote populations increased along a west‐to‐east gradient, with southeastern coyotes being intermediate to western and northeastern coyotes. Similarly, principal component analysis of body mass and linear body measurements suggested that southeastern coyotes were intermediate to western and northeastern coyotes in body size but exhibited shorter tails and ears from other populations. Genetic analyses indicated that southeastern coyotes represented a distinct genetic cluster that differentiated strongly from western and northeastern coyotes. We postulate that southeastern coyotes experienced lower immigration from western populations than did northeastern coyotes, and over time, genetically diverged from both western and northeastern populations. Coyotes colonizing eastern North America experienced different selective pressures than did stable populations in the core range, and we offer that the larger body size of eastern coyotes reflects an adaptation that improved dispersal capabilities of individuals in the expanding range.

## 1 INTRODUCTION

Species commonly respond to shifting selective pressures caused by environmental heterogeneity by exhibiting morphological and genetic variability across their geographic ranges (Fine, 2015; Gould & Johnston, 1972; Mayr, 1970; Sexton, McIntyre, Angert, & Rice, 2009). In return, morphological, genetic, and behavioral divergence among populations may reduce gene flow and facilitate speciation. Therefore, understanding how geographic variation, the basis of genetic variation, originates and which species traits are subject to geographic variation can be of great scientific importance. This understanding requires investigating how geographic variation results from the fit between phenotype and environment and how spatial differences in genetics and morphology translate into population‐level differences (Sexton et al., 2009).

Species ideal for studying geographic variation should occur over broad geographic areas encompassing a range of climates and exhibit substantial variation in morphological and genetic traits. Coyotes (*Canis latrans*) have existed in North America since the Pleistocene (Nowak, 1979, 2002; Tedford, Wang, & Taylor, 2009), currently occupy most biomes of North America (Hody & Kays, 2018), and are considered to be one of the more phenotypically and genetically variable canids (Nowak, 1979; vonHoldt, Cahill et al., 2016; Way, 2007). Although their pre‐Columbian Holocene range included the central and western regions of North America from 55° to 20°N (Hody & Kays, 2018; Jackson, 1951; Nowak, 1979, 2002), the presence of coyotes in eastern North America during the Pleistocene (Nowak, 2002; Tedford et al., 2009) indicates that coyotes have a history of range expansions and contractions that may be attributed to emergence and loss of other *Canis* competitors (Meachen & Samuels, 2012; Nowak, 2002), and changes in climate and landscapes (Koblmüller, Wayne, & Leonard, 2012; Van Valkenburgh, 1999). The arrival of coyotes in eastern North America during the 20th century has generated much interest because it occurred in multiple colonization routes and resulted in noticeable changes in phenotype and hybridization with remnant wolf (*C. lupus, C. lycaon,* and *C. rufus*) populations (Heppenheimer, Cosio et al., 2018; Hody & Kays, 2018; Kays, Curtis, & Kirchman, 2010; Nowak, 2002; Way & Lynn, 2016). Much of this interest has been driven to understand the coyote's ability to adapt to large‐scale, anthropogenic alterations to the landscape, accurately describe their ecological niche, and understand how hybridization with wolves facilitated coyote adaptation to novel habitats in eastern North America (Ellington & Murray, 2015; Kays et al., 2010; Otis, Thornton, Rutledge, & Murray, 2017).

### 1.1 Colonization of eastern North America

**Figure 1:**
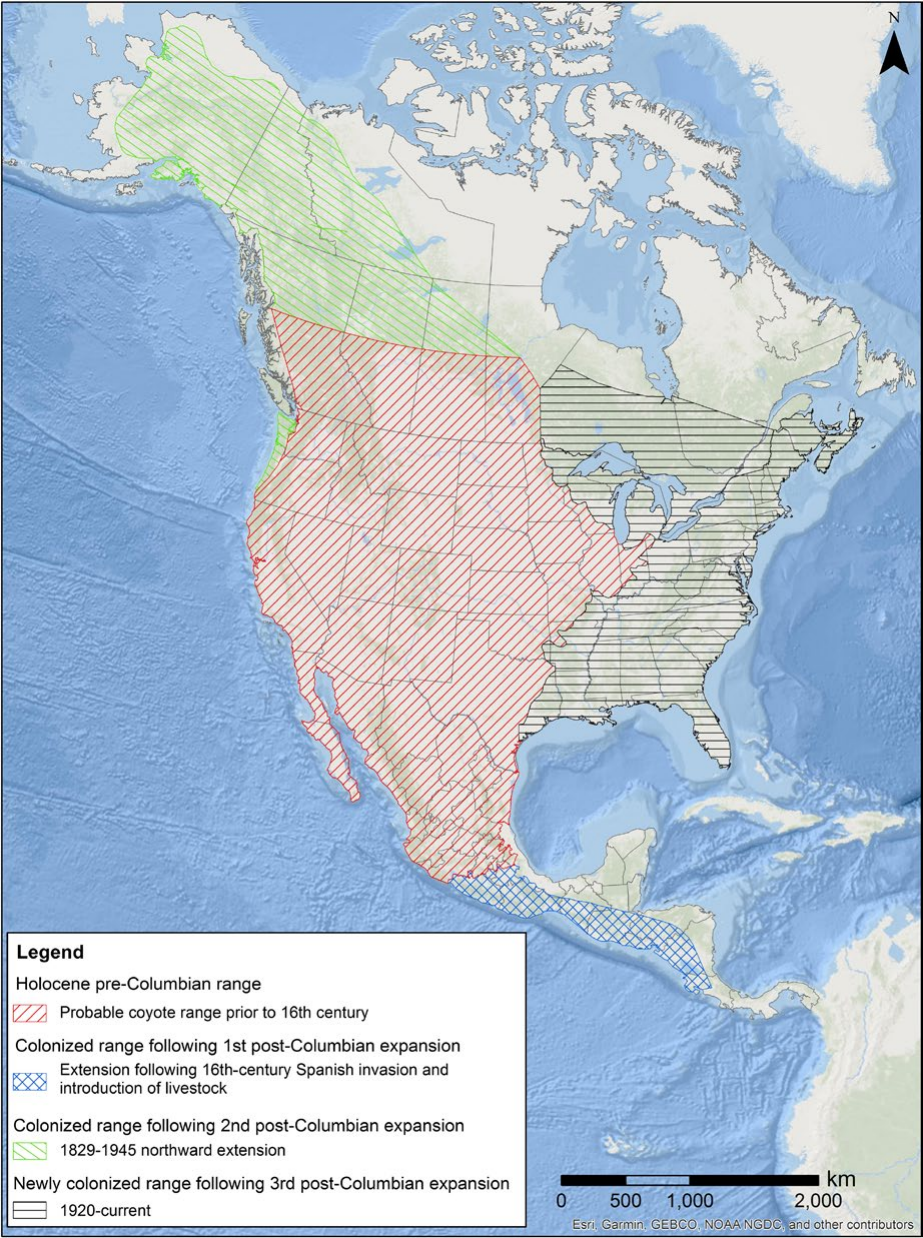
Map of the progressive expansion of the coyote's present distribution from 1685 to 2018

The recent range expansion by coyotes in North America appears to have occurred across three independent expansion events after European colonization (Hody & Kays, 2018; Nowak, 2002; Young, 1951; Figure 1). The initial post‐Columbian coyote range expansion event occurred when coyotes expanded their southern range from central Mexico into Central America during the 16th century following the introduction of cattle to the region by the Spanish (but see Hidalgo‐Mihart, Cantú‐Salazar, González‐Romero, & López‐ González, 2004). The second event was a northward expansion by coyotes from western and central Canada into the Yukon and Alaska that coincided with the gold rushes of the late 19th century. The final and last expansion event occurred in eastern North America during the 20th century in two spatially isolated fronts that began simultaneously during the early 1900s.

Unlike the coyote's previous range expansions, its spread into eastern North America during the 20th century is well described because coyote occurrence was commonly documented at local scales (Hody & Kays, 2018; Nowak, 1979, 2002). The easternmost range of coyotes during the pre‐Columbian Holocene until about 1900 followed the Prairie Peninsula east of the Mississippi River through Illinois, southern Wisconsin, and northern Indiana (Cory, 1912; Hody & Kays, 2018; Jackson, 1961; Mumford, 1969; Nowak, 2002; Young, 1951). After 1900, coyotes from that region moved eastward across the Great Lakes Region into eastern Canada and New England. There is no evidence that coyotes occurred east of the prairies farther south, as Bailey (1905) reported that coyotes were rare east of the semiarid mesquite region that extended eastward as far as north‐central Texas. After 1900, coyotes from the western and central regions of Oklahoma and Texas, and others from northern Missouri, moved into the eastern regions of Oklahoma and Texas and into Alabama, Arkansas, Louisiana, Mississippi, and Tennessee before colonizing the Gulf and Atlantic regions of the Southeast (Nowak, 1979, 2002). Both eastern fronts later converged in the mid‐Atlantic region during the later 20th century (Bozarth, Hailer, Rockwood, Edwards, & Maldonado, 2011; Heppenheimer, Cosio et al., 2018; Hody & Kays, 2018).

### 1.2 Regional designations

Although eastern coyote populations are morphologically and genetically distinct from their western counterparts (Heppenheimer, Brzeski, Hinton et al., 2018; Kays et al., 2010; Nowak, 1979; Way, 2007), there is no clear delineation between eastern and western populations. Recently, Hody and Kays (2018) confirmed that earlier range descriptions by Young (1951) and Nowak (1979) were correct in stating that coyote distribution during the 1800s did not extend into forested ecoregions of the eastern United States. Transition zones between the Great Plains and the eastern temperate forests region occur in at least seven American states and, if these transition zones potentially represent boundaries between western and eastern coyote populations, natural resources agencies for these states are managing two variants of coyotes. Nevertheless, studies typically delimit intraspecific boundaries among coyote populations (e.g., Sacks, Bannasch, Chomel, & Ernest, 2008; Kays et al., 2010; Stronen et al., 2012; Way, 2013; Way & Lynn, 2016) and populations are routinely classified arbitrarily by researchers as western, mid‐Atlantic, midwestern, southwestern, southeastern, or northeastern with little consistency among studies.

Herein, we recognize verified distributional limits of coyotes circa 1900 to separate western and eastern coyote populations along the Great Plains and eastern temperate forests ecoregions (Hody & Kays, 2018; Nowak, 1979; Young, 1951). Recently, Heppenheimer, Brzeski, Hinton et al. (2018) conducted a comprehensive genomewide survey of coyote populations across much of the contiguous United States and southeastern Canada and reported three distinct genetic clusters with one cluster corresponding to the pre‐Columbian Holocene range, a second cluster corresponding to the northeastern expansion range, and a third cluster corresponding to the southeastern expansion range. They also observed moderately high frequencies of intermediate ancestry assignments in the mid‐Atlantic region (e.g., North Carolina, Kentucky, Virginia, Pennsylvania), consistent with previous studies that reported a secondary contact zone between northeastern and southeastern populations existed in mid‐Atlantic (Bozarth et al., 2011; Heppenheimer, Cosio et al., 2018). In agreement with previous research (Nowak, 1979, 2002), Heppenheimer, Brzeski, Hinton et al. (2018) also suggested that western coyote populations in Missouri, Oklahoma, Nebraska, and Texas served as source populations for southeastern coyotes. Therefore, we used 36°30′N, the northern extent of Arkansas, Tennessee, and North Carolina, to separate southeastern and northeastern coyotes in this study, as this likely represents the secondary contact zone reported by previous research.

### 1.3 Coyotes in the southeastern United States

During the mid‐20th century, research on *Canis* ecology in the southeastern United States focused on discriminating among coyote, red wolf, and hybrid populations in Texas, Oklahoma, Arkansas, and Louisiana because it was feared that the last red wolf populations were going extinct through introgressive swamping of coyote genes (McCarly, 1962; Nowak, 1970; Paradiso, 1965; Pimlott & Joslin, 1968). Once red wolves were declared extinct in the wild (USFWS, 1989), research focused on describing the morphological and genetic structure of coyotes in the western regions of the Southeast (Hamilton & Kennedy, 1986; Lydeard & Kennedy, 1988; Lydeard, Leberg, & Baumgardner, 1986; Peppers, Kennedy, & Kennedy, 1996). As coyotes had not established populations along the Atlantic Coast until the turn of the 21st century, there has been a limited amount of research on morphological and genetic characteristics of southeastern coyotes, making it difficult to accurately compare southeastern coyotes to western and northeastern coyotes. Regardless, it has been argued that coyotes in the southeastern United States are a more typical variant of western coyotes comprising small amounts of wolf and dog introgression (Adams, Leonard, & Waits, 2003; vonHoldt, Kays, Kays, Pollinger, & Wayne, 2016; Way & Lynn, 2016; Wheeldon & Patterson, 2017).

There continues to be reports of large canids in rural areas of the Southeast where coyotes replaced red wolves, suggesting that coyotes in the region were morphologically and genetically altered through hybridization with wolves (Giordano & Pace, 2000; Mech & Nowak, 2010). Genetic influence of red wolves, as expressed in the morphology of coyotes, has remained in some areas of the region (Heppenheimer, Brzeski, Wooten et al., 2018; Murphy, Adams, Cox, & Waits 2018). For example, Giordano and Pace (2000) assessed morphometrics of coyote‐like canids at Sabine National Wildlife Refuge, Louisiana, and found that coyotes on the refuge were larger than other Louisiana coyotes, but smaller than red wolves. Indeed, mean body mass of male (20.2 kg) and female (17.6 kg) coyotes reported in Giordano and Pace (2000) was similar to those reported by Hinton and Chamberlain (2014) for genetically identified male (17.8 kg) and female (16.3 kg) red wolf–coyote hybrids.

In theory, genetic introgression may provide novel genotypes selected for in response to new environments and niches (Anderson & Stebbins, 1954; Arnold, 1992; Arnold & Kunte, 2017; Hamilton & Miller, 2016). A widely accepted explanation for the regional shift in coyote body size is that hybridization with wolves introduced adaptive variation that contributed to larger size, which allowed eastern coyotes greater use of white‐tailed deer (*Odocoileus virginianus*; Kays et al., 2010; Power et al., 2015; Way & Lynn, 2016; vonHoldt, Kays et al., 2016). Although hybridization can provide adaptive variation to coyotes, deer populations were mostly extirpated from the southeastern United States when coyotes began colonizing the region during the mid‐20th century (McCabe & McCabe, 1984; VerCauteren, 2003). Indeed, approximately 50,000 white‐tailed deer from Wisconsin and Texas were introduced throughout the Southeast during 1930–1960 to restore deer populations in the region (Adams & Hamilton, 2011; McDonald & Miller, 2004). If phenotypic characteristics in coyote populations resulted from adaptive genetic introgression closely reflecting local adaptations, then characteristics of southeastern coyotes should reflect adaptations to landscapes with low prey availability (e.g., low deer densities) and low interspecific competition (e.g., lower coyote densities) that existed during the mid‐20th century.

### 1.4 Study objectives

Our objective was to describe patterns in morphology and genetics of southeastern coyotes and compare morphometrics and genetics of these coyotes to those from other regions. We believe that examining the whole pattern of geographical variation in a suite of morphological and genetic traits may provide interesting insight into the complexity of geographic variation in coyotes and help develop hypotheses that best explain differences observed between western and eastern populations. For example, body mass has not traditionally been used to delineate subspecific boundaries of coyotes (Jackson, 1951; Nowak, 1979), but mass is the most common phenotypic trait observed and reported by recent studies assessing differences between western and eastern coyotes (Thurber & Peterson, 1991; Gompper, 2002; Way, 2007, 2013; but see Kays et al., 2010). Also, recent genomic research (vonHoldt, Cahill et al., 2016; vonHoldt et al., 2011) suggested that hybridization with dogs may have affected eastern coyote morphology, as evident by black coat color variants in eastern coyotes. Therefore, concomitant with morphometrics, microsatellite data allow us to explore the contribution of dog introgression to eastern coyote morphology. Regardless, variation in morphological and genetic traits resulting from the interplay of geographic and ecological factors has important consequences for key population characteristics, such as reproduction, density, and dispersal. Such differences between eastern and western coyotes have played an important role in stimulating debates regarding ecology, evolution, and conservation of North American *Canis* species (Hohenlohe et al., 2017; Kyle et al., 2006; Rutledge, Devillard, Boone, Hohenlohe, & White, 2015; Rutledge, Wilson, Klütsch, Patterson, & White, 2012; vonHoldt, Cahill et al., 2016; vonHoldt et al., 2017; Way & Lynn, 2016; Wilson et al., 2000).

## 2 MATERIALS AND METHODS

### 2.1 Morphometric analysis

We compiled body mass and linear body measurements of coyotes from two sources. From our first source, we compiled body measurements from ongoing and past projects in the southeastern United States (Alabama, Georgia, Mississippi, North Carolina, South Carolina, and Tennessee) conducted by the authors. Coyotes for these projects were captured using foothold traps (Victor #1.5 and #3 Softcatch, Woodstream Corporation, Lititz, Pennsylvania, USA) with offset jaws. Animals were typically restrained using a catchpole, muzzle, and hobbles, but some were chemically immobilized with an intramuscular injection of ketamine HCl and xylazine HCl to inspect inside the mouth for injuries. We determined sex and weight, and estimated age by tooth wear (Gier, 1968; Gipson, Ballard, Nowak, & Mech, 2000). We categorized coyotes ≥2 years old as adults, 1–2 years old as juveniles, and <1 year old as pups. Animal handling methods followed guidelines approved by the American Society of Mammalogists (Sikes, Gannon, & the Animal Care and Use Committee of the American Society of Mammalogists 2016) and were approved by the University of Georgia Institutional Animal Care and Use Committee (A2014 08‐025‐R2).

Postcranial measurements included body length (anterior tip of the nose pad to the tail base), tail length (tip of the fleshy part of the tail to the tail base), hind foot length (hock to the tip of the digital pads), and shoulder height (tip of the scapula to tip of the digital pads). Cranial measurements included length of head (edge of the premaxillary to the most posterior point of the occipital bone), width of head (most widely separated points), and ear length (edge of the external auditory canal to the tip of the ear). Although these projects used similar anatomical reference points, measurements were recorded from live coyotes by multiple biologists and trappers under varying field conditions that undoubtedly introduced inconsistencies to our dataset. The most obvious inconsistencies involved the length of head and width of head measurements. For example, some projects recorded length of head by measuring the length from the premaxillary to the most posterior point of the occipital bone, whereas others recorded the length from the edge of the nose pad to the most posterior of the occipital bone. To address this problem, we replaced the linear measurements of length of head and width of head in our analyses with a head length to width ratio (length divided by width). Geometric shape expressed by ratios is invariant for a particular measure of size and provides important descriptions of traits without loss of information (Klingenberg, 2016; Mosimann, 1970).

From our second source, we obtained body mass and linear body measurements for coyotes throughout the entire distribution range, using several literature databases (ISI, Google Scholar, JSTOR), literature cited in papers already reviewed (“snowball” sampling), and drawing from our own archives of publications, books, theses, dissertations, and technical reports (Table S1). Because there was no selection bias in our criteria, we believe this approach did not introduce any systematic bias. We included studies in our analyses if they provided, at minimum, body mass of ≥15 coyotes and presented within‐group (e.g., sex, age) means. Because body traits and reference points used to measure them in studies varied, we compiled linear body measurements from other studies if they corresponded with traits measured from the authors’ past and ongoing projects, as noted above.

We then combined mean body mass and linear body measurements for individual coyotes obtained from the literature (2nd data source) with averages calculated from our ongoing and past projects (1st data source), to create a dataset of mean morphometric values for coyote populations sampled across their current range. To evaluate regional differences, we fit a linear mixed‐effects model (LMER) using the statistical software R (R Development Core Team 2014) for comparing mean body mass and linear body measurements among the 3 populations. The LMER included mean body mass and linear body measurements as the response variable, and regions as the explanatory variable with random error structures to account for repeated sampling within U.S. states and Canadian provinces. We then used a type III analysis of variance (ANOVA) to provide inference on the parameters of our LMER. When differences were significant, pairwise comparisons were made using Tukey's range tests.

We combined body mass and linear body measurements for individual coyotes obtained from the literature (2nd data source) with measurements collected from our ongoing and past projects (1st data source), to create a multivariate morphometric dataset. Because multivariate morphometric datasets typically contain a great deal of redundancy, we used a principal component analysis (PCA; JMP software; SAS Institute) to compress this highly dimensional dataset into a lower dimensional one to extract the dominant, underlying gradients of variation (principal components; Gotelli & Ellison, 2004). The principal components (PCs) are weighted linear combinations of the original variables ordered according to the amount of variation each PC explained. We logarithmically transformed our data, as body mass was measured on a different scale than linear body measurements.

For our PCA, we addressed the issue of missing values within our morphometrical dataset by using the restricted maximum‐likelihood (REML) method to create a completed dataset to perform the PCA (Peng & Paul, 2009). The REML method uses a single imputation model to replace missing values with unbiased estimators within the bounds of the existing data. In doing so, REML allowed for missing value uncertainty to be incorporated into our PCA (Peng & Paul, 2009). We used the latent root criterion (PCs with eigenvalues >1) as a stopping rule to determine the number of significant PCs to retain and interpret (McGarigal, Cushman, & Stanford, 2000). We then based our interpretation of each PC on those variables with loadings ≥0.40 or ≤ −0.40 and placed most emphasis on those with loadings ≥0.60 or ≤ −0.60 (McGarigal et al., 2000). We used variables with the strongest loadings to interpret the ecological meaning of each PC.

### 2.2 Genetic analysis

We obtained genetic samples for analyses from tissue (e.g., blood, ear, liver, tongue) of coyotes (*n* = 283) collected from 18 states during 2001–2015 that were within the current coyote range in the United States (Table S2). In a minority of cases, sampling year was unknown but presumed to fall within this approximate period. We collected ear tissue and blood samples from animals captured during some research projects responsible for our first dataset, whereas other tissue was contributions opportunistically collected from hunters and trappers, including 202 previously published samples (Heppenheimer, Cosio et al., 2018; Table S3). Government organizations, such as Florida Fish and Wildlife Conservation Commission, Ohio Department of Natural Resources, United States Department of Agriculture, and United States Fish and Wildlife Service, archived most samples (Princeton University IACUC #1961A‐13). The remaining samples were obtained from the New York State Museum (NYSM‐zm14641; NYSM‐zm15534; NYSM_13643) and the Museum of Southwestern Biology (MSB:Mamm:142883; NK103336, MSB:Mamm:156770; NK154356, MSB:Mamm:230707, MSB:Mamm:231525, MSB:Mamm:265339, MSB:Mamm:265659, MSB:Mamm:273966). In addition to coyote samples, 40 domestic dog samples (blood or buccal swabs) comprising 12 distinct breeds were donated by dog owners (Table S3).

To conduct microsatellite genotyping on all 283 canid samples, DNA was extracted using the DNeasy Blood and Tissue kit (Qiagen, Louisville, KY, USA). DNA was quantified by Qubit 2.0 Fluorometer (Thermo Fisher Scientific, Carlsbad, CA, USA) and standardized between 2 and 5 ng/µl. Each sample was genotyped at 10 microsatellite loci: FH2001, FH2004, FH2010, FH2137 (Francisco, Langston, Mellersh, Neal, & Ostrander, 1996), FH2611, FH2658, FH3399 (Guyon et al., 2003), Pez11, Pez16, and Pez17 (Neff et al., 1999). Similar to Heppenheimer, Cosio et al. (2018), PCRs were a total volume of 10 µl and contained 1.5 µl (3–10 ng) DNA, 5.0 µl Type‐It master mix (Qiagen), 2.1 µl ddH_2_O, 0.4 µl 10 mg/ml BSA (New England Biolabs, Ipswich, MA), and 1.0 µL of primer mix, which included a forward primer with a 5′ 16 bp‐M13F sequence tag, a 6‐FAM‐labeled complement to the M13F tag (Boutin‐Ganache, Raposo, Raymond, & Deschepper, 2001) and an unlabeled reverse primer. Cycling conditions included an initial denaturation at 95°C for 15 min, followed by 25 cycles at 94°C for 30 s, 59°C for 90 s, and 72°C for 60 s, then 15 cycles at 94°C for 30 s, 53°C for 90 s, and 72°C for 60 s, with a final extension at 60°C for 30 min. We included 22 randomly selected positive controls that amplified ≥3 times to confirm consistent genotyping across PCRs. To ensure our reagents were not contaminated, we included water controls with each reaction. We denatured PCR products with Hi‐Di formamide (Applied Biosystems, Foster City, CA, USA) and LIZ GeneScan 500 size standard (Applied Biosystems), and the resulting PCR fragments were analyzed on an ABI 3730XL capillary sequencer. Genotypes were manually called in GENEIOUS v6.1.6 (Kearse et al., 2012). We removed samples with more than 30% missing data prior to analysis.

We calculated standard summary statistics, including observed and expected heterozygosity, linkage disequilibrium (LD), and deviations from Hardy–Weinberg equilibrium (HWE) at all coyote sampling locations (i.e., states) and within dogs, with ARLEQUIN v3.5.2.2 (Excoffier & Lischer, 2010). Pairwise *F*_ST_ between all sampling locations and geographic regions were also calculated with ARLEQUIN. Additionally, we calculated average number of alleles per locus with GenAlEx v6.503 (Peakall & Smouse, 2006, 2012) and allelic richness (*A*_R_) using the R package hierfstat (Goudet, 2005).

We conducted analyses of population structure of the 243 coyotes and 40 domestic dogs in STRUCTURE v2.3.4 (Pritchard, Stephens, & Donnelly, 2000). Using the admixture model and no prior population assumptions, we conducted 10 runs for each *K *(1–10) with 500,000 repetitions after a burn‐in of 250,000. We combined results from each independent run with CLUMPP v64.1.1.2 (Jakobsson & Rosenberg, 2007). We evaluated optimal number of genetic clusters represented by the data by considering both the log‐likelihood (LnProbability) values calculated via STRUCTURE (Pritchard et al., 2000) and the Evanno Method (Δ*K*) (Evanno, Regnaut, & Goudet, 2005), which was implemented with STRUCTURE HARVESTER v0.6.94 (Earl & vonHoldt, 2012). We considered individuals admixed if ancestry proportions (i.e., *Q* values) were <0.8 for any given inferred cluster (Heppenheimer, Cosio et al., 2018).

We evaluated the association of pairwise and geographic distances to assess the extent of isolation by distance among our sample populations with a series of Mantel tests implemented in the R package ade4 (Dray & Dufour, 2007). Pairwise genetic distances were calculated as normalized *F*_ST_ (Rousset, 1997). We calculated pairwise geographic distances between sampling locations as the shortest straight‐line distance between state centroids using the Advanced Google Maps Distance Calculator (Daft Logic 2017).

## 3 RESULTS

**Figure 2:**
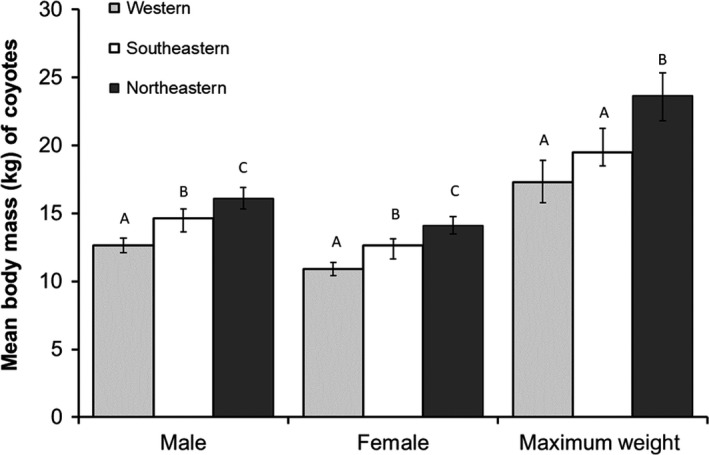
Mean body mass of coyote populations of the continental United States. Letters above the bars represent statistical differences among regions within male, female, and maximum weight categories (*p* < 0.05, Tukey's test)

As few measurements of linear body dimensions were reported in studies, we only compared mean body mass among coyote populations (Table S1). The LMER model ANOVA indicated a statistically significant difference among coyote populations for male (*F*_2,32_ *= *20.652, *p* < 0.001) and female (*F*_2,32_ *= *28.332, *p* < 0.001) body mass. Mean (±*SD*) body mass reported for male northeastern coyotes was 16.2 kg (±1.2) and was greater than those reported for southeastern (14.7 kg ± 2.1) and western (12.7 kg ± 1.2) coyotes (Figure 2). Similarly, mean body mass reported for female northeastern coyotes was 14.3 kg (±1.1) and was greater than those reported for southeastern (12.6 kg ± 1.8) and western (11.0 kg ± 0.9) coyotes (Figure 2). The LMER model ANOVA indicated that maximum body mass reported for northeastern coyotes averaged 23.0 kg (±2.0) and was greater than southeastern (19.5 kg ± 1.2) and western (17.3 kg ± 3.2) coyotes (*F*_2,23_ *= *13.551, *p* < 0.001), whereas no difference was observed between western and southeastern coyotes (Figure 2).

**Figure 3:**
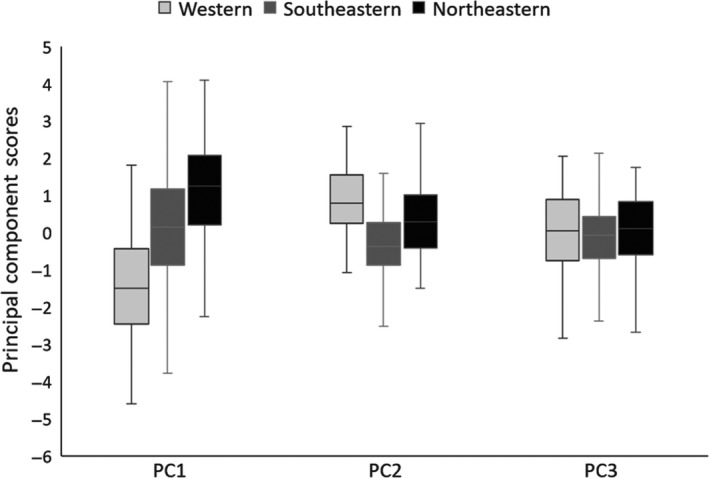
Mean principal components scores for PC1 (body size), PC2 (appendage lengths), and PC3 (head dimensions) of western, southeastern, and northeastern coyotes

For our PCA, we assessed the measurements of 481 coyotes from northeastern (12.1%), southeastern (70.6%), and western (17.3%) populations. The first three principal components (PC1, PC2, and PC3), which explained 43.1%, 17.7%, and 16.1% of the cumulative variation, respectively, were the only PC scores with eigenvalues >1 (Table 1). The eigenvalues of PC1 comprised positive loadings for body mass, body length, hind foot length, and shoulder height, whereas eigenvalues for PC2 comprised negative loadings for body length and positive loadings for ear and tail lengths (Table 1). The eigenvalues of PC3 comprised positive loadings for ear length and head length to width ratio. Collectively, these PC scores indicate that once PC1 accounted for body size, PC2 and PC3 accounted for variation in appendage lengths and head dimensions, respectively. Mean PC1 (body size) scores for southeastern coyotes were intermediate to those for western and northeastern coyotes (*F*_2,478_ *= *41.795, *p* < 0.001; Figure 3). Mean PC2 (appendage lengths) scores for southeastern coyotes were less than those for western and northeastern coyotes (*F*_2,478_ *= *54.770, *p* < 0.001; Figure 3). Mean PC3 (head dimensions) scores for coyotes were similar for all regions (*F*_2,478_ *= *0.512, *p* = 0.600; Figure 3).

**Table 1:** Eigenvalues, share of total variance along with eigenvectors, and factor loadings of body measurements recorded from coyotes in western, northeastern, and southeastern regions of the United States

Body measurements	Principal component 1		Principal component 2		Principal component 3	
	Eigenvector	Loading	Eigenvector	Loading	Eigenvector	Loading
Body mass	0.51	0.88	−0.09	−0.10	−0.09	−0.10
Ear length	0.21	0.37	0.62	0.69	0.47	0.50
Tail length	0.32	0.56	0.40	0.45	−0.30	−0.31
Body length	0.37	0.66	−0.53	−0.59	−0.01	−0.01
Hind foot length	0.47	0.81	0.20	0.22	0.10	0.10
Shoulder height	0.48	0.83	−0.18	−0.20	−0.17	−0.18
Head length: head width ratio	0.11	0.19	−0.30	−0.33	0.80	0.85
Eigenvalue	3.02		1.24		1.13	
% of total variance	43.07		17.74		16.13	
Description	Body size		Appendage lengths		Head dimensions	

We observed high genetic diversity across all coyote populations (average *H*_e_ = 0.84) and slightly lower diversity within dogs (*H*_e_ = 0.80). Following Bonferroni correction, we observed no significant deviations from HWE across coyote populations (α = 0.05, *p* > 2.6 × 10^−4^), but we did observe significant deviations from HWE at two loci within dogs (FH2004, *p* = 7.0 × 10^−5^; Pez16, *p* = 6.0 × 10^−5^). Further, following Bonferroni correction we observed significant LD between two additional loci in Louisiana coyotes (FH2001 & FH3399; *p* < 5.85 × 10^−5^). Removal of these loci from Louisiana coyotes produced similar results in downstream analyses (data not shown). We also observed significant linkage between nine loci pairs within dogs. However, these deviations from HWE and LD within dogs are attributable to selective breeding processes associated with domestication and unlikely to have significantly biased results. As such, removal of dogs did not impact coyote cluster assignments in the population structure analysis (data not shown).

When coyotes were analyzed by region, the greatest genetic differentiation was observed between the northeastern and southeastern populations (*F*_ST_ = 0.022, *p* < 1 × 10^−5^). Furthermore, southeastern coyotes were more genetically differentiated from western coyotes (*F*_ST_ = 0.018; *p* < 1 × 10^−5^) than northeastern coyotes were (*F*_ST_ = 0.013; *p* < 1 × 10^−5^). Coyotes sampled from the eastern contact zone (North Carolina, Virginia) were not included in these calculations.

**Figure 4:**
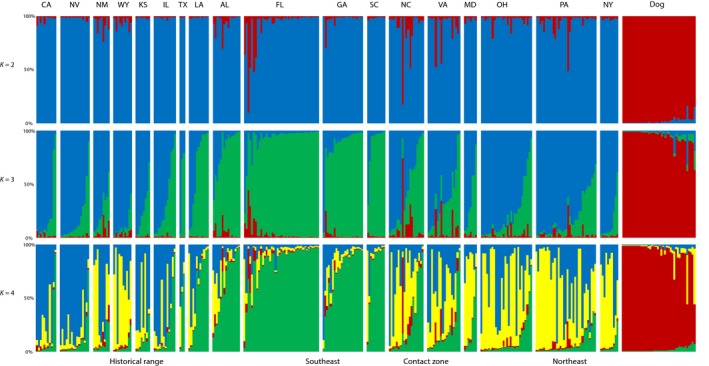
Genetic structure inferred by Bayesian clustering in STRUCTURE at *K* = 2, *K* = 3, and *K* = 4 with sampling locations indicated on the x‐axis

In our analysis of population structure, when two populations were assumed (*K = *2), assignments to inferred clusters corresponded to species designations. Despite this clear separation of coyotes and dogs, a minority of coyotes were considered admixed, with ancestry proportions <0.8 for either inferred cluster, and two coyote samples from Florida and North Carolina clustered strongly with the dog population (*Q*_Dog_ > 0.8). When three populations were assumed (*K = *3), which was the optimal number of clusters, coyote populations were further separated by geographic location (Supporting Information Figure S1; Table S4). One major cluster consisted of southeastern coyotes, and the other cluster consisted of western and northeastern coyotes (Figure 4). Similar to clustering patterns at *K = 2*, a minority of coyote samples had intermediate assignments to the dog cluster (*Q*_Dog_ > 0.2). Of these admixed samples, one originated from western coyotes (New Mexico), four originated from southeastern coyotes (Alabama, Florida, Georgia), one originated from northeastern coyotes (Pennsylvania), and five originated from coyotes in the eastern contact zone (Heppenheimer, Cosio et al., 2018). At *K = 4*, 36 coyotes exhibited a high assignment to the new cluster (Figure 4). Most (*n* = 24) of these samples were northeastern coyotes. The remaining samples were western coyotes (*n* = 5), southeastern coyotes (*n* = 2), and coyotes in the eastern contact zone (*n* = 5). Despite this weak large‐scale population structure, we observed a weak but nonsignificant correlation between genetic and geographic distance among southeastern coyote populations (Mantel test: *r* = 0.465, *p* = 0.159; Supporting Information Figure S2). However, there did appear to be significant isolation by distance among western and northeastern coyote populations (Mantel test: *r* = 0.267, *p* = 0.019; Supporting Information Figure S2).

## 4 DISCUSSION

Phenotypic responses to the environment are common and can be observed through patterns of geographic variation (Fine, 2015; Gould & Johnston, 1972; Mayr, 1970). Coyotes historically occupied a large geographic range across North America prior to European colonization, and their recent range expansion may have facilitated population divergence in peripheral populations established in eastern North America (see discussion among Kays & Monzón, 2017; Way & Lynn, 2016; Wheeldon & Patterson, 2017). Our results showed that mean body mass of coyote populations increased across a west‐to‐east gradient, a trend reported in other studies (Kays et al., 2010; Thurber & Peterson, 1991; Way, 2007). Furthermore, our PCA suggested that southeastern coyotes were intermediate in body size to western and northeastern coyotes, exhibited shorter ear and tail lengths, but did not have narrower or shorter heads. Finally, our genetic analysis indicated that southeastern coyotes represent a unique genetic cluster, suggesting these coyotes are more genetically distinct from western coyotes than northeastern coyotes are from western coyotes.

**Figure 5:**
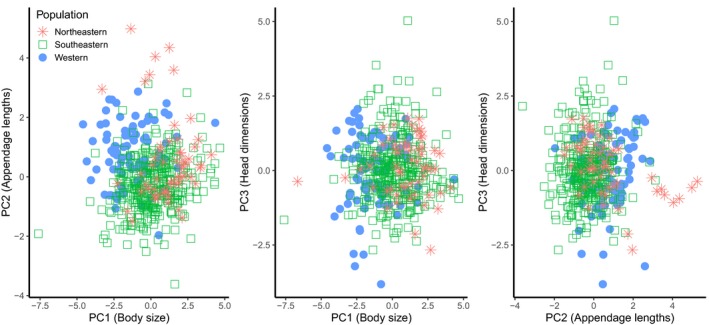
Scatter plots of 3 between‐group principal components of the principal component analysis

**Figure 6:**
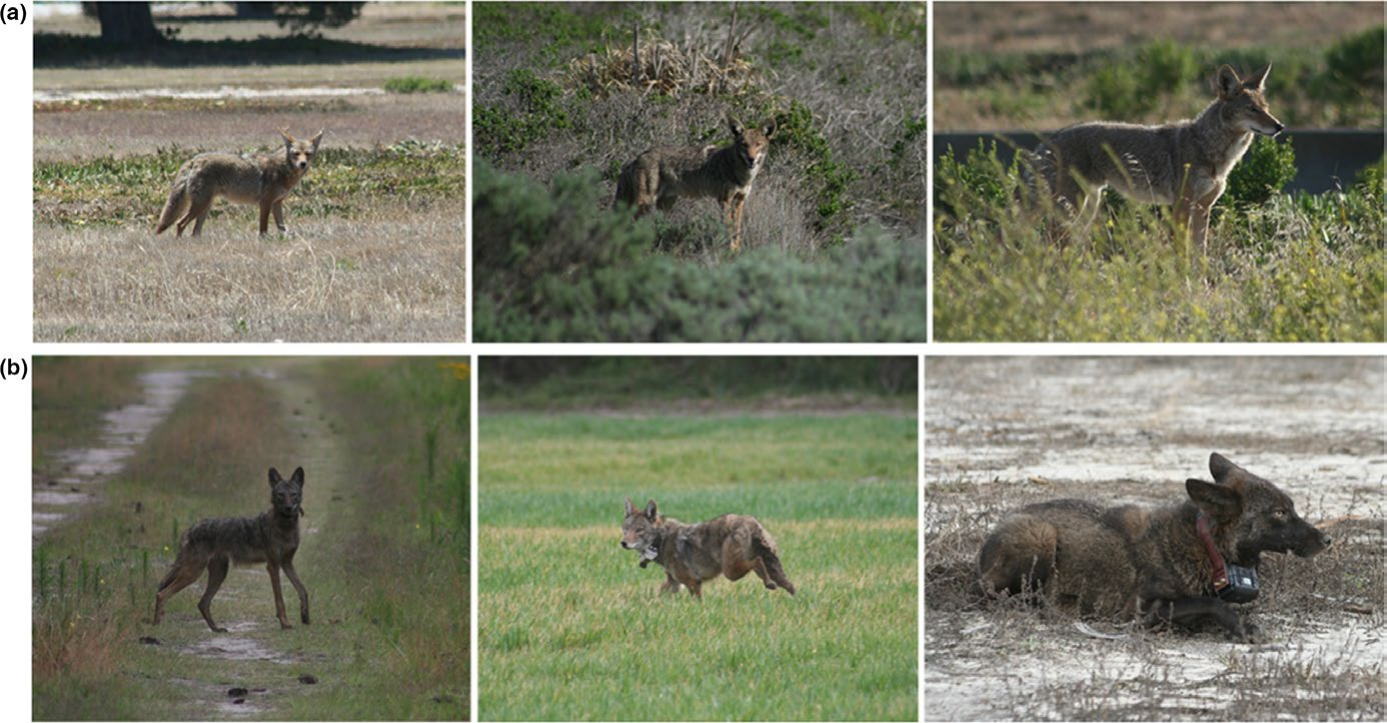
Photographic comparison of western and southeastern coyotes. Photograph credit and location as follows: (a) Western coyotes, Santa Barbara County, California, credit: J. Hinton. (b) Southeastern coyote, Hyde County, North Carolina, credit: J. Hinton. Southeastern coyote, Washington County, North Carolina, credit: J. Hinton. Melanistic southeastern coyote, Saluda County, South Carolina, credit: J. Hinton

When we removed the effect of body size (PC1), we found that appendage lengths (PC2) and head dimensions (PC3) were important traits accounting for the remaining variation explained by our PCA. This is not surprising because ear, tail, and skull morphologies do not exhibit the same allometric relationship observed for the axial and appendicular skeleton that are more influenced by weight bearing (Carter, 1987; Reynolds, 2002; Wang & Tedford, 2010). We interpreted our PCA results to mean that southeastern coyotes typically have smaller ears and shorter tails than do northeastern and western coyotes but, in all three regions, head dimensions did not appear to be proportionally different (Figures 5 and 6). Ear and tail morphologies vary among canid species and can be used to differentiate canid taxa (Cavallini, 1995; Hinton & Chamberlain, 2014; Sillero‐Zubiri, Hoffmann, & Macdonald, 2004). Although both are known to play important roles in canid communication (Lehner, 1978), ear length is associated with thermoregulation (Feldhamer, Drickamer, Vessey, Merritt, & Krajewski, 2015; Geffen & Girard, 2003; Maloiy, Kamau, Shkolnik, Meir, & Arieli, 1982; Sillero‐Zubiri et al., 2004) and enlarged ears in canids can enhance low‐frequency hearing in open environments (Wang & Tedford, 2010). Similar to previous studies (Jackson, 1951; Nowak, 1979), we observed that ear and rostrum lengths were variable among regions and suggest that variable hearing and olfactory adaptations are plausible for coyote populations inhabiting a wide continuum of habitats from open deserts and grasslands to heavily forested regions of North America and should be further investigated. Additionally, tail morphology is understudied and underappreciated in ecological studies of canids but has an important influence on locomotion qualities (e.g., bursting, running, jumping, balance; Hickman, 1979) and, similar to ear length, may be an adaptation to changes in habitat structure and other environmental factors.

Skull morphology is strongly associated with feeding adaptations, and craniodental characters (e.g., shape, dentition, biting force) can influence resource use and structure carnivore communities (Davies, Meiri, Barraclough, & Gittleman, 2007; Donadio & Buskirk, 2006; Rosenzweig, 1966; Van Valkenburgh, 1988). Although our measurement of head dimensions could not detect finer structural differences (e.g., dentition, dome of head, frontal sinus), we believe similarity in head dimensions among regions likely reflects a general similarity in diet among coyote populations in all three regions, as changes in shape (i.e., short vs. long) would indicate adaptation to differing stresses related to feeding ecology (Curtis, Orke, Tetradis, & Valkenburgh, 2018; Van Valkenburgh, 1991; Wang & Tedford, 2010). Regardless of region, studies of coyote diets typically report consistent use of lagomorphs, small mammals and, to a lesser extent, ungulates while exhibiting variable use of fruit (Carrera et al., 2006; Clark, 1972; Hernández, Parmenter, Dewitt, Lightfoot, & Laundré., 2002; Hinton, Ashley et al., 2017; Lingle & Pellis, 2002; Patterson, Benjamin, & Messier, 1998; Todd & Keith, 1983; Ward et al., 2018). Therefore, we suggest that a broader inclusion of craniodental and postcranial measurements, and when possible genetic markers, be used to sufficiently investigate to what extent differences exist geographically among coyote populations and what selection pressures may influence variation in coyote morphology (e.g., Murray & Boutin, 1991; Kays et al., 2010; Curtis et al., 2018).

Our microsatellite analysis reported low levels of population differentiation for coyotes within the three geographic regions, but a notable differentiation among regions. Specifically, we observed two major genetic clusters that separated southeastern coyotes into one population whereas northeastern and western coyotes were separated into the second population. This finding contradicts previous studies suggesting that southeastern coyotes were genetically more representative of western coyotes than northeastern coyotes were (Adams et al., 2003; Kays et al., 2010; Way & Lynn, 2016). Although our analysis of population structure only loosely resolved northeastern and western coyotes, we did observe a weak but significant correlation between genetic distance and geographic distance between sampling locations in these populations, suggesting measurable genetic differences. Moreover, we observed low levels of dog ancestry in southeastern and northeastern coyotes, and admixed individuals did not appear to be more common in southeastern coyotes. Therefore, our results do not support the conclusion that strong separation of southeastern coyotes from all other sampled locations is the result of extensive interbreeding with dogs. The pattern of low regional differentiation could have resulted from the coarse resolution of our dataset and weak discrimination power of our set of microsatellite markers to detect genetic variation at finer scales. Although we believe our results to be robust and informative, as we have analyzed more coyotes from a broader geographic range than previous studies (e.g., Adams et al., 2003; Bohling et al., 2017; Bozarth et al., 2011; Damm, Armstrong, Arjo, & Piaggio, 2015; Kays et al., 2010; Roy, Geffen, Smith, Ostrander, & Wayne, 1994; Way, Rutledge, Wheeldon, & White, 2010), we suggest that future studies with small sample sizes use genomewide markers, such as restriction site‐associated DNA sequencing (RADseq), to document finer population structure and stronger patterns of isolation by distance than microsatellites (Vendrami et al., 2017). Regardless, the stronger affinity between western and northeastern coyotes than between western and southeastern coyotes is a novel observation that may clarify the evolutionary and demographic past of southeastern coyotes.

The distribution of genetic variation can be influenced by migration rates between populations (Bell & Gonzalez, 2011; Eckert, Samis, & Lougheed, 2008; Sexton et al., 2009) and observed differences between southeastern coyote populations and those from other regions may have resulted from founder effects, genetic drift, and local adaptations due to reduced immigration to peripheral populations that became established in the southeastern United States during 1900–1960. Clearly, coyote colonization of eastern North America occurred along two distinct expansion routes that began simultaneously and experienced introgressive hybridization with wolves but exhibited different rates of movement and gene flow (Bozarth et al., 2011; Heppenheimer, Cosio et al., 2018; Kays et al., 2010). Therefore, it is plausible that colonization of the Northeast benefitted from the presence of stable western coyote populations in the Prairie Peninsula (Cory, 1912; Jackson, 1961; Mumford, 1969), which extends east of the Mississippi River through Illinois, southern Wisconsin, and northern Indiana. However, colonization of the Southeast was hampered by the extirpation of coyotes in parts of central and eastern Texas via massive poisoning programs to protect sheep from 1900 to 1950 (Bailey, 1907; Gabrielson, 1936; Nowak, 1979; Russell & Shaw, 1971). A large “canid free” zone adjacent to the Southeast achieved some temporary break in coyote populations that may have bottlenecked immigration of western coyotes to the Southeast through Oklahoma, Missouri, and Arkansas and forced coyotes to recolonize large regions of Texas before expanding to the Southeast. With reduced immigration from western coyote populations, newly established populations of southeastern coyotes were less connected to their western counterparts than were northeastern populations.

When species expand their ranges, populations along edges of expansion fronts experience new selective pressures on reproductive and dispersal traits that stable populations do not (Bell & Gonzalez, 2011; Burton, Phillips, & Travis, 2010; Gaston, 2009; Sexton et al., 2009). In particular, research shows that peripheral populations on range edges typically exist at lower densities and increase investment for greater dispersal ability (Burton et al., 2010; Phillips, Brown, Travis, & Shine, 2008; Travis & Dytham, 2002). Therefore, we suggest that increased body sizes observed in eastern coyote populations were induced by hybridization (Kays et al., 2010; Nowak, 1979, 2002; Power et al., 2015) and larger coyotes were then favored over smaller coyotes in the expansion range because larger coyotes had greater dispersal capabilities that improved immigration among peripheral populations. Coyote populations consist of a significant proportion of transient individuals (Hinton, Manen, & Chamberlain, 2015; Kamler & Gipson, 2000; Morin & Kelly, 2017; Windberg & Knowlton, 1988), and recent research suggests that transiency is an important life‐history strategy that facilitates metapopulation dynamics (Hinton et al., 2015) and regulates population densities (Morin & Kelly, 2017) via compensatory immigration. The probability of surviving transiency and finding suitable habitat and mates in the expansion range may have been greater for larger‐bodied coyotes because they had greater movement radii and fasting endurances than did smaller individuals (Lindstedt & Boyce, 1985; McCue, 2010; Millar & Hickling, 1990). Likewise, coyotes appear poorly adapted to hunting in forested habitats (Crête, Ouellet, Tremblay, & Arsenault, 2001; Richer, Crête, Ouellet, Rivest, & Huot, 2002; Thibault & Oullett, 2005) and increased body weights of offspring would lower the risk of starvation and reproductive failure in the eastern forests of North America. For those reasons, we propose that the larger body size observed in eastern coyotes reflects an adaptation to increase dispersal and reproductive capabilities on the expansion range rather than greater reliance on white‐tailed deer, a species that was extirpated or reduced in abundance from most areas of the southeastern United States when coyotes began colonizing the region (McCabe & McCabe, 1984; VerCauteren, 2003).

When describing geographical patterns of intraspecific variation in coyotes, understanding how selective forces act on characters and the genetic basis for phenotypic variation is crucial. Although the coyote genome and resulting phenotypes are shaped by natural forces, it is widely acknowledged that humans have influenced local and regional genotypes by altering landscapes, extirpating larger competitors, and facilitating hybridization between coyotes and wolves in eastern North America. Indeed, research has shown that human‐mediated mortality of wolves disrupts the social structure of wolf packs and reduces their abundance on the landscape (Borg, Brainerd, Meier, & Prugh, 2015; Milleret et al., 2017) which allowed coyotes to colonize regions formerly held by wolves and hybridize with surviving individuals (Hinton, Brzeski, Brzeski, Rabon, & Chamberlain, 2017; Rutledge, Patterson et al., 2010). Introgression typically extends into the range of the receding species (Rohwer, Bermingham, & Wood, 2001; Secondi, Faivre, & Bensch, 2006; Steeves, Maloney, Hale, Tylianakis, & Gemmell, 2010), and asymmetrical introgression from coyotes began as they invaded ranges of the declining eastern wolf and red wolf (Nowak, 2002; Rutledge, Loveless, Loveless, & Patterson, 2010). If wolf genes were adaptive in coyotes, they spread in the invading coyote population and became rapidly fixed in the gene pool following demographic growth (Currat, Ruedi, Petit, & Excoffier, 2008). Although it is commonly asserted that increases observed in body sizes of eastern coyotes were an adaptation for greater reliance on white‐tailed deer (Kays et al., 2010; Power et al., 2015; vonHoldt, Kays et al., 2016; Way & Lynn, 2016), it is difficult to reconcile how greater use of deer would benefit key population characteristics (i.e., reproduction and dispersal) more in eastern coyote populations than in western populations because western coyotes are also known to prey on ungulates (Bleich, 1999; Gese & Grothe, 1995; Keller, Millspaugh, Lehman, & G., & Mong, T. W., 2013; Lingle & Pellis, 2002). Rather, we suggest that coyotes on the expansion front in eastern North America experienced different selective pressures than did stable populations in the core range, and it is plausible that increased body sizes of eastern coyotes reflect adaptations that improved dispersal abilities of individuals in the expanding range. For example, Heppenheimer, Brzeski, Hinton et al. (2018) reported that three genes (*CACNA1C, ALK,* and *EPHA6*) known to have putative functions related to dispersal were more associated with eastern coyotes than western coyotes. Therefore, we suggest that selective pressure on the eastern expansion range favored larger coyotes because of their greater dispersal capabilities, rather than their ability to kill deer. Clearly, increased dispersal distances would have improved connectivity among metapopulations of coyotes in eastern North America during the colonization period of the mid‐20th century.

## CONFLICT OF INTEREST

None declared.

## AUTHOR CONTRIBUTIONS

J.W.H. conceived the project, designed the study, organized and did field work, data analysis, and drafted the manuscript; E.H. contributed to the project design, organized and did laboratory work, genetic analysis, contributed intellectually, and drafted the manuscript; K.M.W. contributed intellectually, assisted with data analysis and drafting the manuscript, and edited/approved the manuscript; D.C. contributed intellectually, provided morphometric data, and edited/approved the manuscript; M.L.K. contributed intellectually, provided GIS help, and edited/approved the manuscript; J.C.K. contributed intellectually, provided morphometric data, and edited/approved the manuscript; J.M. organized and did field work; provided morphometric data, and edited/approved the manuscript; K.V.M. contributed intellectually, secured funding, and edited/approved the manuscript; M.W. contributed intellectually, organized and did field work, provided morphometric data, and edited/approved the manuscript; B.vH. contributed to the project design, secured funding, provided equipment, contributed intellectually, and edited/approved the manuscript; M.J.C. contributed to the project design, secured funding, provided equipment, contributed intellectually, and edited/approved the manuscript.

## Data Availability

Data available from the Dryad Digital Repository: http://doi:10.5061/dryad.742cc92.
