# Supplementary material for: Geographic patterns in morphometric and genetic variation for coyote populations with emphasis on southeastern coyotes
Source: Ecol Evol. 2019 Feb 21;9(6):3389–404. doi: 10.1002/ece3.4966 (PMC6434562; doi:10.1002/ece3.4966)
Supplement: Supplementary file 1 [file ECE3-9-3389-s001.docx]

Table S1: Reported mean body masses (kg) of coyotes from studies in the United States and Canada.

| State/Province | Population | N | Males | Females | Max^a^ | Source |
| --- | --- | --- | --- | --- | --- | --- |
| Alabama | Southeastern | 24 | 16.0 | 13.8 | 18.0 | Authors |
| Alaska | Western | 54 | 12.9 | 11.1 |  | Thurber and Peterson (1991) |
| Alberta | Western | 39 | 12.1 | 11.5 | 15.0 | Bowen 1982 |
| Alberta | Western | 26 | 12.3 | 10.6 | 16 | Murray 2014 |
| Arizona | Western | 19 | 14.3 | 10.8 |  | Danner 1976 |
| Arizona | Western | 185 | 11.1 | 9.4 |  | Fisher 1980 |
| Arizona | Western | 17 | 10.5 | 8.8 |  | Witham 1977 |
| Arkansas | Southeastern | 175 | 15.0 | 13.0 |  | Gipson 1978 |
| California | Western | 73 | 12.1 | 10.2 |  | Cypher 1995 |
| California | Western | N/A^b^ | 11.2 | 9.8 |  | Hawthorne 1971 |
| California | Western | 19 | 12.2 | 11.6 |  | Shoemaker 1985 |
| California | Western | 25 | 12.0 | 10.1 |  | Lyren 2001 |
| Florida | Southeastern | 218 | 13 | 11.1 | 19.1 | Authors |
| Illinois | Western | 977 | 14.1 | 12.1 | 22.3 | Nelson and Lloyd 2005 |
| Iowa | Western | N/A | 13.4 | 11.4 | 17.4 | Andrews and Boggess 1978 |
| Iowa | Western | N/A | 13.0 | 11.0 |  | Gier 1968 |
| Kansas | Western | 596 | 13.1 | 10.9 | 20.4 | Boggess and Henderson 1977 |
| Kansas | Western | N/A | 14.1 | 11.8 |  | Gier 1968 |
| Louisiana | Southeastern | 18 | 20.4 | 17.4 | 21.8 | Giordano 2000 |
| Maine | Northeastern | 59 | 15.9 | 14.5 |  | Hilton 1976 |
| Maine | Northeastern | 48 | 15.8 | 13.7 |  | Richens and Hugie 1974 |
| Maine | Northeastern | 60 | 17.3 | 15.5 | 20.5 | Harrison 1986 |
| Maine | Northeastern | 47 | 16.6 | 14.2 |  | Wilson et al. 2004 |
| Massachusetts | Northeastern | 35 | 17.9 | 16.0 | 25.1 | Way 2007 |
| Minnesota | Western | 204 | 12.5 | 11.5 |  | Berg and Chesness 1978 |
| Minnesota | Western | 69 | 12.8 | 11.4 |  | Mech and Paul 2008 |
| Minnesota | Western | N/A | 14 | 12 |  | Gier 1968 |
| Mississippi | Southeastern | 57 | 14.1 | 12.6 | 19.9 | Authors |
| Nevada | Western | 20 | 12.3 | 11.9 | 16 | Authors |
| New Brunswick | Northeastern | 86 | 14.6 | 13.1 |  | Dumond and Villard 2000 |
| New Brunswick | Northeastern | 73 | 16.6 | 15.2 |  | Moore and Millar 1986 |
| New Mexico | Western | 34 | 11.6 | 9.5 |  | Windberg et al. 1997 |
| New Mexico | Western | 829 | 11.1 | 10.1 |  | Young 1951 |
| New York | Northeastern | 163 | 16.1 | 14.7 |  | Goff et al. 1984 |
| New York | Northeastern | 19 | 14.2 | 11.9 |  | Brundige 1993 |
| North Carolina | Southeastern | 116 | 14.1 | 13.1 | 18.2 | Hinton and Chamberlain 2014 |
| Nova Scotia | Northeastern | 39 | 18.0 | 14.6 |  | Power et al. 2015 |
| Nova Scotia | Northeastern | 89 | 16.5 | 13.7 | 25.9 | Parker 1995 |
| Nova Scotia | Northeastern | 129 | 15.4 | 12.6 | 21.7 | Sabean 1993 |
| Ohio | Western | 40 | 14.8 | 13.4 | 20.7 | Wallace 2013 |
| Oklahoma | Southeastern | 120 | 14.9 | 12 | 20.9 | Freeman and Shaw 1979 |
| Oklahoma | Southeastern | N/A | 14.7 | 12.1 |  | Litvaitis 1978 |
| Ontario | Northeastern | 82 | 17.3 | 14.7 | 22.8 | Wheeldon and Patterson 2012 |
| Pennsylvania | Northeastern | 131 | 16.5 | 14.5 | 23.2 | Authors |
| Prince Edward Island | Northeastern | 86 | 16.5 | 14 |  | Field 2003 |
| Prince Edward Island | Northeastern | 90 | 16 | 15 | 25 | Parker 1995 |
| Quebec | Northeastern | 49 | 16 | 14.1 |  | Poulle et al. 1995 |
| Quebec | Northeastern | 19 | 13.6 | 12.5 |  | Wilson et al. 2004 |
| Quebec | Northeastern | 27 | 13.9 | 12.1 |  | Hout et al. 1995 |
| Rhode Island | Northeastern | 36 | 16.6 | 15.3 | 21.4 | Way 2007 |
| Savannah River Area^c^ | Southeastern | 218 | 14.2 | 12.6 | 20 | Authors |
| Savannah River Area | Southeastern | 32 | 14.3 | 11.7 | 18.8 | Schrecengost 2007 |
| South Dakota | Western | 26 | 11.8 | 10.6 | 13.0 | Chronert 2007 |
| Tennessee | Southeastern | 67 | 13.5 | 12.2 | 19.8 | Authors |
| Tennessee | Southeastern | 55 | 14.0 | 11.9 |  | Stephenson and Kennedy 1993 |
| Texas | Western | 16 | 14 | 11.9 |  | Kamler 2002 |
| Texas | Western | 93 | 13.2 | 11.5 |  | Meinzer and Guthery 1980 |
| Texas | Western | 67 | 10.9 | 10 |  | Meinzer et al. 1975 |
| Texas | Southeastern | 135 | 11.4 | 9.6 |  | Windberg et al. 1991 |
| Texas | Western | 84 | 12.6 | 10.5 |  | Young 1951 |
| Vermont | Northeastern | 42 | 16.9 | 14.5 | 25 | Lorenz 1978 |
| Vermont | Northeastern | 17 | 17.8 | 16.6 | 21.4 | Person 1988 |
| Virginia | Southeastern | 70 | 16.2 | 13.4 |  | Houben and Mason 2004 |
| West Virginia | Southeastern | 39 | 14.4 | 11.9 |  | Wykle 1999 |
| Yellowstone National Park | Western | N/A | 13.6 | 11.8 |  | Reported in Way 2007 |

^a^Maximum body mass reported in study, ^b^sample size not reported but assumed ≥ 15 coyotes, ^c^measurements recorded from coyotes from 10 counties along the Georgia and South Carolina border.

Table S2: Diversity statistics across 10 microsatellite loci for 283 coyotes and 40 domestic dogs. (Abbreviations: sample size, n; observed heterozygosity, H_O_; expected heterozygosity, H_e_; average number of alleles per locus, A_N_; Allelic richness, A_R_).

| **Sampling Location** | **n** | **H_o_** | **H_e_** | **A_N_** | **A_R_** |
| --- | --- | --- | --- | --- | --- |
| **Northeastern Expansion** | | | | | |
| Maryland | 7 | 0.84 | 0.82 | 6.30 | 4.02 |
| New York | 10 | 0.83 | 0.80 | 6.60 | 4.10 |
| Ohio | 28 | 0.86 | 0.85 | 11.00 | 4.29 |
| Pennsylvania | 33 | 0.83 | 0.84 | 11.00 | 4.15 |
| Overall | 78 | 0.84 | 0.83 | 8.73 | 4.14 |
| **Contact Zone** | | | | | |
| North Carolina | 19 | 0.86 | 0.85 | 10.20 | 3.89 |
| Virginia | 18 | 0.83 | 0.86 | 9.30 | 4.30 |
| Overall | 37 | 0.85 | 0.85 | 9.75 | 4.10 |
| **Southeastern Expansion** | | | | | |
| Alabama | 15 | 0.85 | 0.83 | 9.20 | 4.23 |
| Florida | 41 | 0.86 | 0.85 | 11.40 | 4.24 |
| Georgia | 22 | 0.83 | 0.83 | 10.20 | 4.24 |
| Louisiana | 11 | 0.86 | 0.84 | 7.80 | 4.17 |
| South Carolina | 10 | 0.85 | 0.85 | 7.50 | 4.20 |
| Overall | 99 | 0.85 | 0.84 | 9.22 | 4.21 |
| **Historical Range** | | | | | |
| California | 11 | 0.80 | 0.85 | 7.90 | 4.24 |
| Illinois | 12 | 0.83 | 0.81 | 8.20 | 4.06 |
| Kansas | 8 | 0.84 | 0.85 | 7.50 | 4.36 |
| Nevada | 16 | 0.82 | 0.83 | 9.00 | 4.30 |
| New Mexico | 7 | 0.81 | 0.85 | 6.30 | 4.26 |
| Texas | 3 | 0.83 | 0.87 | 4.30 | 4.30 |
| Wyoming | 12 | 0.83 | 0.86 | 9.30 | 4.42 |
| Overall | 69 | 0.82 | 0.85 | 7.50 | 4.28 |
| **Dog** | | | | | |
| Dog | 40 | 0.65 | 0.80 | 9.10 | 3.90 |

Table S4.—Average Q-values per state inferred via STRUCTURE.

| **State** | **Average Q_Dog_** | **Average Q_Southeast_** | **Average Q_Coyote_** |
| --- | --- | --- | --- |
| California | 0.02 | 0.22 | 0.76 |
| Nevada | 0.01 | 0.17 | 0.82 |
| New Mexico | 0.08 | 0.22 | 0.70 |
| Wyoming | 0.03 | 0.09 | 0.88 |
| Kansas | 0.02 | 0.25 | 0.73 |
| Illinois | 0.02 | 0.35 | 0.63 |
| Texas | 0.01 | 0.51 | 0.48 |
| Louisiana | 0.01 | 0.66 | 0.33 |
| Alabama | 0.05 | 0.78 | 0.18 |
| Florida | 0.05 | 0.88 | 0.07 |
| Georgia | 0.03 | 0.84 | 0.13 |
| South Carolina | 0.02 | 0.86 | 0.12 |
| North Carolina | 0.10 | 0.38 | 0.52 |
| Virginia | 0.07 | 0.32 | 0.61 |
| Maryland | 0.03 | 0.37 | 0.61 |
| Ohio | 0.03 | 0.20 | 0.76 |
| Pennsylvania | 0.04 | 0.17 | 0.79 |
| New York | 0.02 | 0.14 | 0.84 |
| Dogs | 0.94 | 0.03 | 0.04 |

Figure S1. Plot of mean likelihood L(K) and variance per K value from STRUCTURE on a dataset containing 283 coyotes from western, northeastern, and southeastern North America genotyped for 10 polymorphic microsatellite loci.


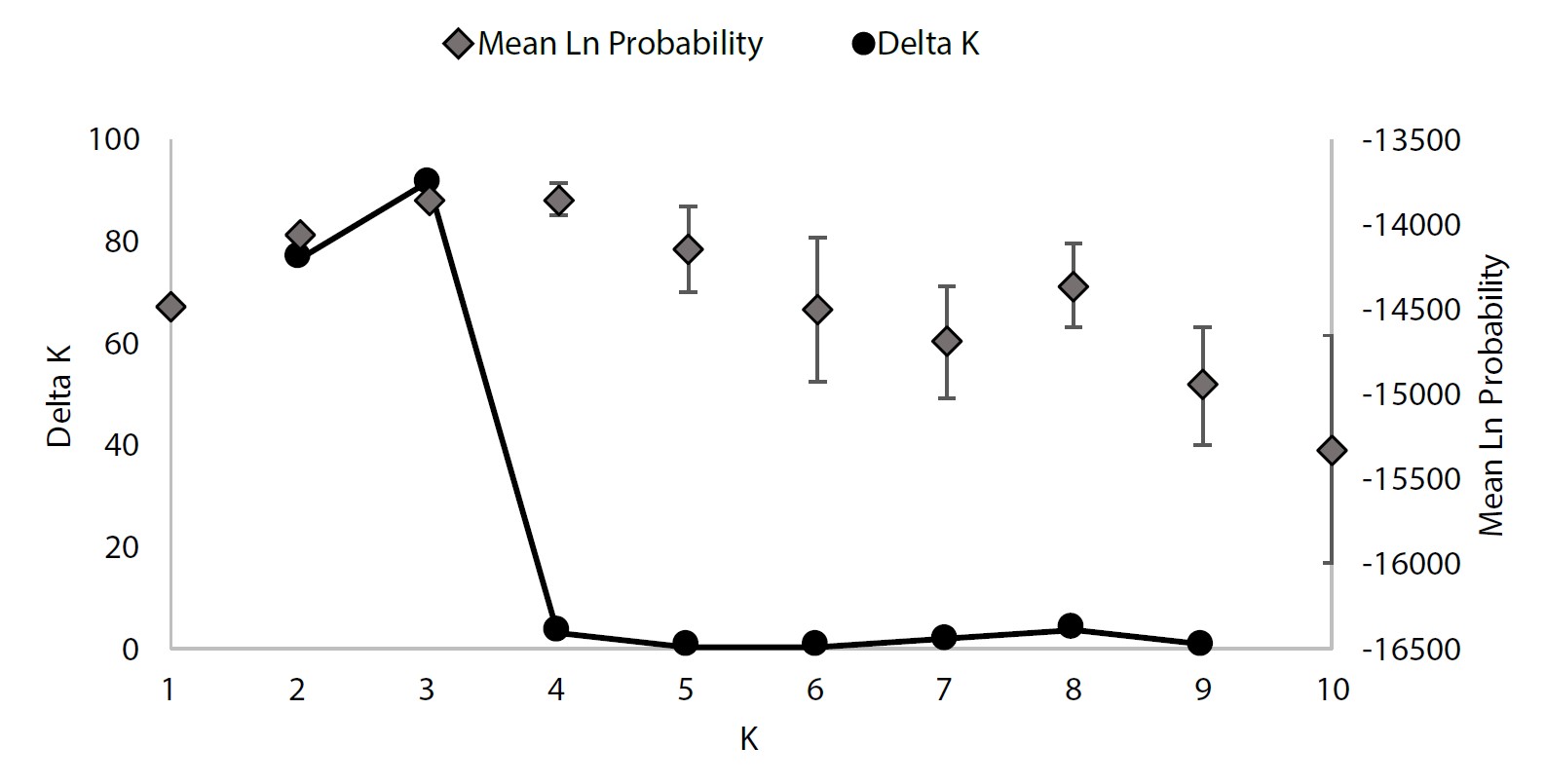


Figure S2. Pairwise genetic distance versus geographic distance between (A) southeastern and (B) all other sampling locations.


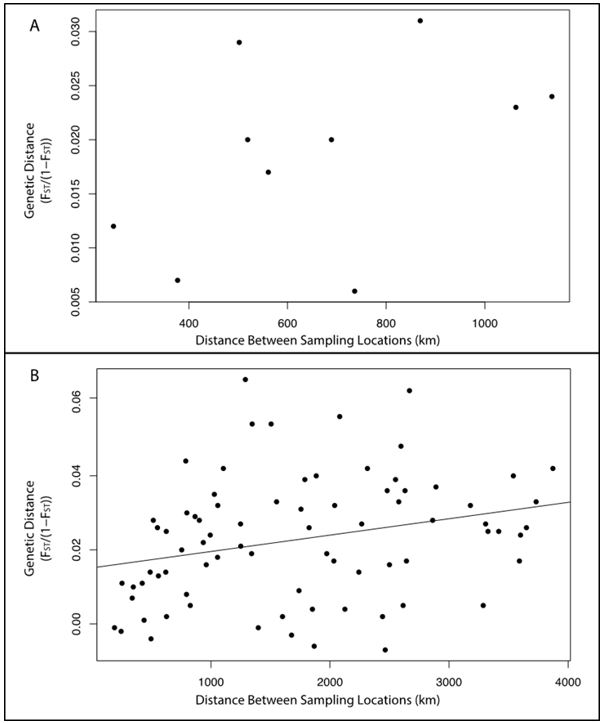


**References**

Andrews, R. D., & Boggess, E. K. (1978). Ecology of coyotes in Iowa. In M. Bekoff (Ed.), *Coyotes: biology, behavior, and management* (pp. 249–265). Caldwell, NJ: Blackburn Press.

Berg, W. E., & Chesness, R. A. (1978). Ecology of coyotes in northern Minnesota. In M. Bekoff (Ed.), *Coyotes: biology, behavior, and management* (pp. 229–247). Caldwell, NJ: Blackburn Press.

Boggess, E. K., & Henderson, F. R. (1977). Regional weights of Kansas coyotes. *Transactions of the Kansas Academy of Science* **80**, 79–80.

Bowen, W. D. (1982). Home range and spatial organization of coyotes in Jasper National Park, Alberta. *Journal of Wildlife Management* **46**, 201–216.

Brundige, G. C. (1993). Predation ecology of the eastern coyote (*Canis latrans* var.) in the Adirondacks, New York. Ph.D. dissertation, State University of New York, Syracuse, New York, USA.

Chronert, J. M. (2007). Ecology of the coyote (*Canis latrans*) at Wind Cave National Park. M.Sc. Thesis, South Dakota State University, Brookings, South Dakota, USA.

Cypher, B. L. (1995). Coyote morphometric characteristics and mass dynamics in the San Joaquin Valley, California. *Southwestern Naturalist* **40**, 360–365.

Danner, D. A. (1976). Coyote home range, social organization, and scent post visitation. M.Sc. Thesis, University of Arizona, Tucson, Arizona, USA.

Dumond, M., & Villard, M-A. (2000). Demography and body condition of coyotes (*Canis latrans*) in eastern New Brunswick. *Canadian Journal of Zoology* **78**, 399–406.

Field, S. A. (2003). Population ecology of eastern coyotes (*Canis latrans*) on Prince Edward Island. M.Sc. Thesis, University of Prince Edward Island, Charlottetown, Prince Edward Island, Canada.

Fisher, A.R. (1980). Influence of an abundant supply of carrion on population parameters of the coyote. Ph.D. dissertation, University of Arizona, Tucson, Arizona, USA.

Freeman, R. C., & Shaw, J. H. (1979). Hybridization in *Canis* (Canidae) in Oklahoma. *Southwestern Naturalist* **24**, 485–499.

Gier, H. T. (1968). Coyotes in Kansas. *Kansas State College Agricultural Experiment Station Bulletin* **393**, 1–118.

Giordano, M. R. (2000). Morphology, movement patterns and habitat use of coyote-like canids in southwest Louisiana. M.Sc. Thesis, Louisiana State University, Baton Rouge, Louisiana, USA.

Gipson, P. S. (1978). Coyotes and related *Canis* in the southeastern United States with a comment on Mexican and Central American *Canis*. In M. Bekoff (Ed.), *Coyotes: biology, behavior, and management* (pp. 191–208). Caldwell, NJ: Blackburn Press.

Goff, G. R., Okoniewski, J. C., McCarty, S. L., & Decker, D. J. (1984). New York’s Wildlife Resources: Eastern coyote (*Canis latrans*). Cornell, NY: Cornell Department of Natural Resources.

Harrison, D. J. (1986). Coyotes in the Northeast. *Appalachia* **182**, 30–39.

Hawthorne, V. M. (1971). Coyote movements in Sagehen Creek Basin, northeastern California. *California Fish and Game* **57**, 154–161.

Hilton, H. (1976). The physical characteristics, taxonomic status, and food habits of the eastern coyote in Maine. M.Sc. Thesis, University of Maine, Orono, Maine, USA.

Hinton, J. W., & Chamberlain, M. J. (2014). Morphometrics of *Canis* taxa in eastern North Carolina. *Journal of Mammalogy* **95**, 855–861.

Houben, J. M., & Mason, J. R. (2004). Weight and age of coyotes captured in Virginia, USA. *Proceedings of the Vertebrate Pest Conference* **21**, 75–76.

Hout, J., Poulle, M. L., & Crête, M. (1995). Evaluation of several indices for assessment of coyote (*Canis latrans*) body composition. *Canadian Journal of Zoology* **73**, 1620–1624.

Kamler, J. F. (2002). Relationships of swift foxes and coyotes in northwest Texas. Ph.D. dissertation, Texas Tech University, Lubbock, Texas, USA.

Litvaitis, J. A. (1978). Movements and habitat use of coyotes on the Wichita National Wildlife Refuge. M.Sc. Thesis, Oklahoma State University, Stillwater, Oklahoma, USA.

Lorenz, J. R. (1978). Physical characteristics, movement, and population estimate of the eastern coyote in New England. M.Sc. Thesis, University of Massachusetts, Amherst, Massachusetts, USA.

Lyren, L. M. (2001). Movement patterns of coyotes and bobcats relative to roads and underpasses into the Chino Hills area of southern California. M.Sc. Thesis, California State Polytechnic University, Pomona, USA.

Mech, L. D., & Paul, W. J. (2008). Wolf body-mass cline across Minnesota: related to taxonomy? *Canadian Journal of Zoology* **86**, 933–936.

Meinzer Jr., W. P., & Guthery, F. S. (1980). Age distributions and weights of coyote in northwestern Texas. *Southwestern Naturalist* **25**, 275–278.

Meinzer, W. P., Ueckert, D. N., & Flinders, J. T. (1975). Food niche of coyotes in the Rolling Plains of Texas. *Journal of Range Management* **28**: 22–27.

Moore, G. C., & Millar, J. S. (1986). Food habits and average weights of a fall-winter sample of eastern coyotes, *Canis latrans*. *Canadian Field-Naturalist* **100**, 105–106.

Murray, M. H. (2014). Individual variation in the ecology of urban coyotes and implications for human-coyote conflict. Ph.D. Dissertation, University of Alberta, Alberta, Canada.

Nelson, T. A., & Lloyd, D. M. (2005). Demographics and conditions of coyotes in Illinois. *American Midland Naturalist* **153**, 418–427.

Parker, G. R. (1995). *Eastern coyote: the story of its success*. Halifax, Nova Scotia: Nimbus Publishing.

Person, D. K. (1988). Home range, activity, habitat use, and food habits of eastern coyotes in the Champlain Valley Region of Vermont. M.Sc. Thesis, University of Vermont, Burlington, Vermont, USA.

Poulle, M. L., Crête, M., & Huot, J. (1995). Seasonal variation in body mass and composition of eastern coyotes. *Canadian Journal of Zoology* **73**, 1625–1633.

Power, J. W. B., LeBlanc, N., Bondrup-Nielsen, S., Boudreau, M. J., O’Brien, M. S., & Stewart, D. T. (2015). Spatial genetic and body-size trends in Atlantic Canada *Canis latrans* (coyote) populations. *Northeastern Naturalist* **22**, 598–612.

Richens, V. B., & Hugie, R. D. (1974). Distribution, taxonomic status, and characteristics of coyotes in Maine. *Journal of Wildlife Management* **38**, 447–454.

Sabean, B. (1993). Coyote carcass collections. *Nova Scotia Trappers Newsletter* **29**, 10.

Schrecengost, J. D. (2007) Home range and food habits of the coyote (*Canis latrans*) at the Savannah River Site, South Carolina. M.Sc. Thesis, University of Georgia, Athens, Georgia, USA.

Shoemaker, S. (1985). Food selection, home range, and movements of coyotes on and off a sanctuary in Klamath Basin. M.Sc. Thesis, Oregon State University, Corvallis, Oregon, USA.

Stephenson, S. W., & Kennedy, M. L. (1993). Demography of a coyote population in western Tennessee. *Journal of the Tennessee Academy of Science* **68**, 122–124.

Thurber, J. M., & Peterson, R. O. (1991). Changes in body size associated with range expansion in the coyote (*Canis latrans*). *Journal of Mammalogy* **72**, 750–755.

Wallace, B. F. (2013). Coyote spatial and temporal use of recreational parklands as a function of human activity within the Cuyahoga Valley, Ohio. M.Sc. Thesis, University of Akron, Akron, Ohio, USA.

Way, J. G. (2007). A comparison of body mass of *Canis latrans* (coyotes) between eastern and western North America. *Northeastern Naturalist* **14**, 111–124.

Wheeldon, T. J., & Patterson, B. R. (2012). Genetic and morphological differentiation of wolves (*Canis lupus*) and coyotes (*Canis latrans*) in northeastern Ontario. *Canadian Journal of Zoology* **90**, 1221–1230.

Wilson, P., Jakubas, J., & Mullen, S. (2004). Genetic status and morphological characteristics of Maine coyotes as related to neighboring coyote and wolf populations. Final report to the Maine Outdoor Heritage Fund Board. Augusta, ME: Maine Department of Inland Fisheries and Wildlife.

Windberg, L. A., Engeman, R. M., & Bromaghin, J. F. (1991). Body size and condition of coyotes in southern Texas. *Journal of Wildlife Diseases* **27**, 47–52.

Windberg, L. A., Ebbert, S. M., & Kelly, B. T. (1997). Population characteristics of coyotes (*Canis latrans*) in the northern Chihuahuan Desert of New Mexico. *American Midland Naturalist* **138**, 197–207.

Witham, J. H. (1977). Movement and spacing patterns of female coyotes near Anderson Mesa, Arizona. M.Sc. Thesis, Northern Arizona University, Flagstaff, Arizona, USA.

Wykle, J. (1999). The status of the coyote, *Canis latrans*, in West Virginia. Ph.D. Dissertation, Marshall University, Huntington, West Virginia.

Young, S. P. (1951). History, life habits, economic status, and control. In S. P. Young, H. H. T. Jackson (Eds.), *The clever coyote* (pp. 1–226). Harrisburg, PA: Stackpole, Company.
